# Analysis of prognostic factors of undifferentiated pleomorphic sarcoma and construction and validation of a prediction nomogram based on SEER database

**DOI:** 10.1186/s40001-022-00810-z

**Published:** 2022-09-15

**Authors:** Zimao Wang, Jinhua Liu, Jingjing Han, Zheng Yang, Qiying Wang

**Affiliations:** Department of Plastic Surgery, First Affiliated Hospital of Zhengzhou University, Zhengzhou, 450052 Henan China

**Keywords:** Undifferentiated pleomorphic sarcoma, Survival, SEER database, Nomogram

## Abstract

**Background:**

Undifferentiated pleomorphic sarcoma (UPS) is considered one of the most common types of soft tissue sarcoma (STS). Current studies have shown that the prognosis of UPS is related to some of its clinical characteristics, but no survival prediction model for the overall survival (OS) of UPS patients has been reported. The purpose of this study is to construct and validate a nomogram for predicting OS in UPS patients at 3, 5 years after the diagnosis.

**Methods:**

According to the inclusion and exclusion criteria, 1079 patients with UPS were screened from the SEER database and randomly divided into the training cohort (*n* = 755) and the validation cohort (*n* = 324). Patient demographic and clinicopathological characteristics were first described, and the correlation between the two groups was compared, using the Kaplan–Meier method and Cox regression analysis to determine independent prognostic factors. Based on the identified independent prognostic factors, a nomogram for OS in UPS patients was established using R language. The nomogram’s performance was then validated using multiple indicators, including the area under the receiver operating characteristic curve (AUC), consistency index (C-index), calibration curve, and decision curve analysis (DCA).

**Results:**

Both the C-index of the OS nomogram in the training cohort and the validation cohort were greater than 0 .75, and both the values of AUC were greater than 0.78. These four values were higher than their corresponding values in the TNM staging system, respectively. The calibration curves of the Nomogram prediction model and the TNM staging system were well fitted with the 45° line. Decision curve analysis showed that both the nomogram model and the TNM staging system had clinical net benefits over a wide range of threshold probabilities, and the nomogram had higher clinical net benefits than the TNM staging system as a whole.

**Conclusion:**

With good discrimination, accuracy, and clinical practicability, the nomogram can individualize the prediction of 3-year and 5-year OS in patients with UPS, which can provide a reference for clinicians and patients to make better clinical decisions.

## Background

Soft tissue sarcomas (STS) are heterogeneous and rare tumors showing mesenchymal differentiation that accounts for less than 1% of all malignant neoplasms, of which undifferentiated pleomorphic sarcoma (UPS) is considered to be one of the most common types of soft tissue sarcoma in adults [1, 2]. In recent years, statistical prediction models such as nomograms have been increasingly used to predict the probability of clinical events, and for clinicians and cancer patients, providing individualized prognostic information is essential for clinical decision-making. The nomogram can convert the complex regression equation into a visual graph, individualize the prediction of the prognosis of cancer patients, and make the survival prognosis results more intuitive and easy to understand, so it has a great application prospect in clinical work. In the past, some researchers collected the clinical information of UPS patients from the SEER database from 1990 to 2015 and established a CSS survival prediction model for UPS patients [3]. Since the SEER database added tumor size, tumor depth and radiotherapy of patients diagnosed after 2004, this study selected UPS patients diagnosed between 2004 and 2015 and collected the clinical data of UPS patients through retrieval to determine the independent prognostic impact. Based on the information above, the study established the OS nomogram survival prediction model for UPS patients. Moreover, the nomogram survival prediction model constructed in this study added three more variables, tumor size, tumor depth and radiotherapy, which can better provide a reference for on-the-spot treatment decisions of UPS.

## Materials and methods

### Research object

#### Data acquisition 

The clinical data of patient with UPS were retrieved from SEER database through SEER*Stat8.3.9 software (http://seer.cancer.gov//seerstat/), including age, sex, race, tumor site, tumor size, tumor depth, T stage, N stage, M stage, tumor grade, surgery, radiotherapy, chemotherapy, survival outcome, survival time, etc. The data in the SEER database are publicly and freely available and has no privacy implications. The application and warranty for use of data had been signed, and we obtained the corresponding login account and permission of data use (username: 19352-Nov2019). Therefore, this study did not need to provide the approval and informed consent of the institutional ethical review board.

#### Inclusion criteria

1. The year of diagnosis was 2004–2015;
2. Histopathological diagnosis in accordance with the International Classification of Diseases Oncology Album Third Edition (ICD-O-3) classification of UPS (8830/3);
3. The primary site was in the subcutaneous soft tissue (Soft Tissue including Heart in ICD-O-3/WHO 2008 site code. Primary Site was limited to the subcutaneous soft tissue, and corresponding code was C49.0-C49.9);

#### Exclusion criteria

**Fig. 1:**
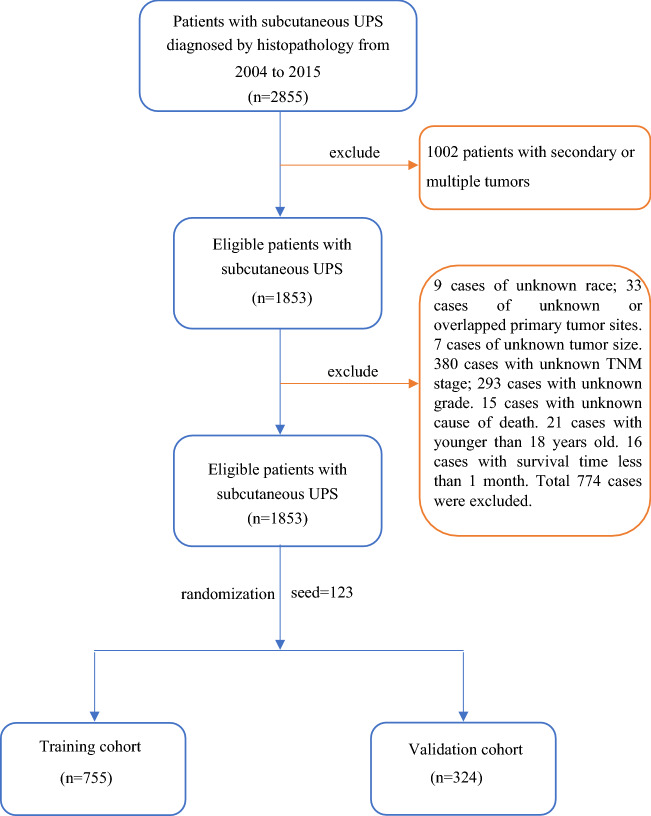
Flowchart of patient screening and grouping

1. Patients with single and non-primary UPS;
2. Patients with incomplete clinical information, such as race, tumor site, tumor size, T stage, N stage, M stage, tumor grade and other unknown clinicopathological information;
3. The source of patient reports was only limited autopsy or death certificate;
4. Patients with unknown cause of death;
5. Patients with age < 18 years old;
6. Patients with survival time < 1 month or patients with incomplete follow-up information;

According to the inclusion and exclusion criteria, a total of 1079 cases were screened from the SEER database and randomly divided into the training cohort (*n* = 755, 70%) and the validation cohort (*n* = 324, 30%) by a random number (seed = 123), and the specific patient screening flowchart is shown in Fig. 1.

### *Selection of *prognostic* factors*

The selection of prognostic factors was related to the establishment of subsequent nomograms. The selection of nomogram variables was not only based on the results of univariate and multivariate Cox regression analysis, but also needs to refer to other relevant literature and clinical experience, considering practical problems such as clinical application [4]. Therefore, based on clinical experience and literature review, the following 13 variables were considered as possible prognostic factors: age, sex, race, tumor site, tumor size, tumor depth, T stage, N stage, M stage, tumor grade, surgery, radiotherapy, chemotherapy.

### Events and indicators of ending

The ending events in this study were death due to various causes. The time from diagnosis of UPS to death due to various causes is called overall survival. The ending indicators were 3-year and 5-year overall survival rates of patients with UPS.

#### Statistical methods 

**Fig. 2:**
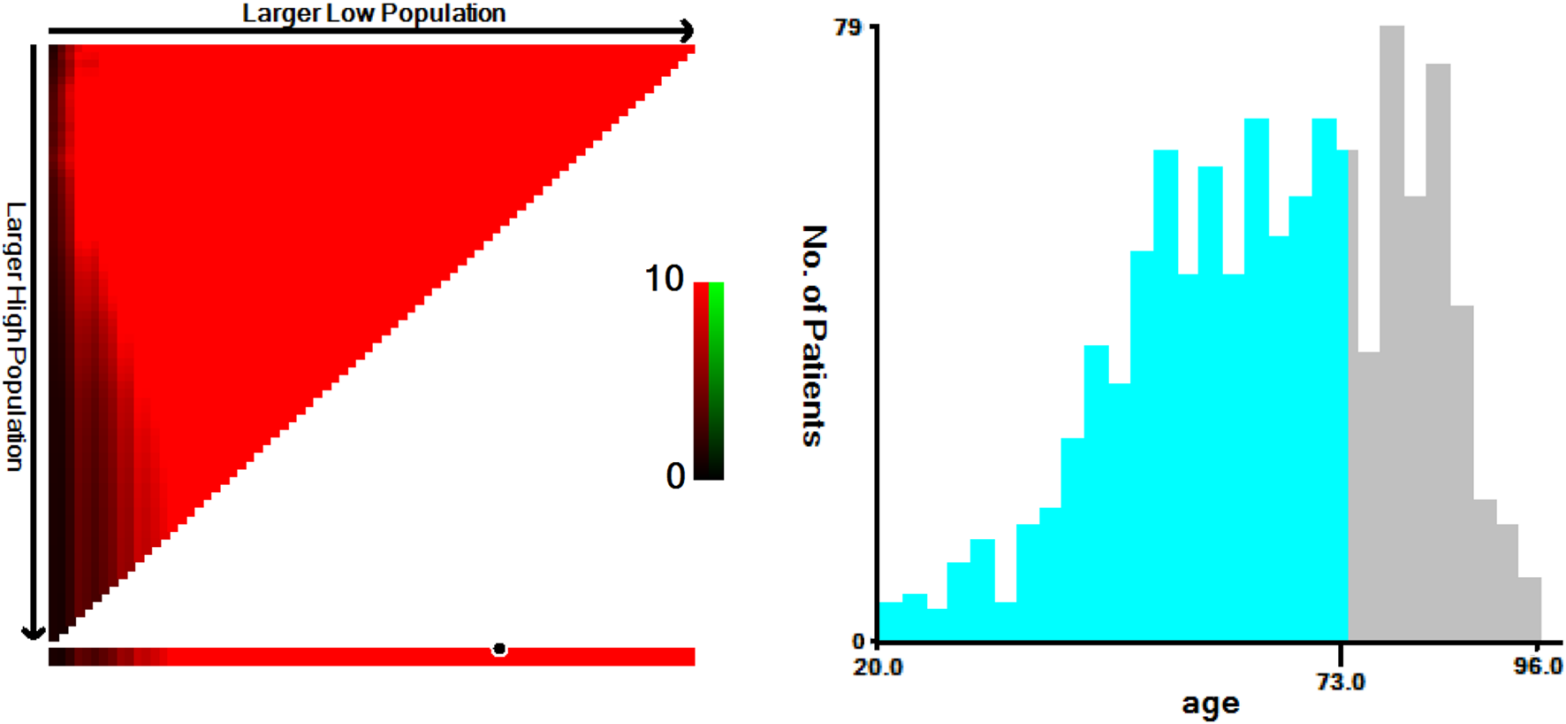
Optimal age cut-off under overall cancer survival

X-tile software (Yale University, New Haven, Connecticut, USA) can convert continuous variables into categorical variables by finding the optimal cut-off values [5]. The optimal cut-off value of age was found by X-tile software, and the patients were divided into two groups: ≤ 73 years old and ≥ 74 years old (Fig. 2). Descriptive statistical analysis was used for demographic and clinical factors, and Chi-square test was used to evaluate the correlation between the training and validation cohorts. Kaplan–Meier method was used to make the survival curves of each factor, and log-rank test was used for comparison. Cox regression analysis was used to identify factors associated with the prognosis of patients with UPS by stepwise backward selection. In the training cohort, univariate and multivariate Cox regression analyses were applied to screen out independent prognostic factors as well as the corresponding hazard ratio (HR) and 95% confidence interval (95% CI).

Based on the identified independent prognostic factors, a survival prediction model based on training cohort was developed through the "rms" package in R language to predict 3- and 5-year OS. The principle of nomogram is that by constructing multivariate regression models (such as Cox regression and logistic regression, etc.), the nomogram score each influencing factor according to the contribution of each influencing factor in the model to the outcome variable (the size of the regression coefficient). Then add each score to the total score. Finally, through the functional conversion relationship between the total score and the probability of outcome events, the predicted value of the individual outcome event is calculated.

Based on the data of the training and the validation cohorts, internal and external validation were performed, respectively, in terms of discrimination, calibration, and clinical practicality. The concordance index (C-index), receiver operating characteristic curve (ROC) and the corresponding area under the curve (AUC) were used to assess the discrimination of the nomogram. The closer the c-index is to 1, the better the discrimination, as is AUC. Generally speaking, when the value is greater than 0.7, the model is proved to be more reliable.

The calibration curve was obtained by repeated sampling 1000 times by Bootstrap self-sampling method to assess the closeness between the predicted value and the actual value, that is, the accuracy of the nomogram model prediction [6]. The principle of the calibration curve is to calculate the predicted survival rate of each patient based on the nomogram, sort the predicted values in ascending order and group them by quartile (or other quantiles), and separately calculate the means of the predicted and the actual (observed) survival rate of each group. An ideal calibration curve is a straight line that goes through the origin with a slope being 1, which is a 45-degree line. The more the calculated calibration curve overlaps the 45-degree line, the better the model matches the patients' actual risk.

Finally, the R language the "gg DCA" package performs decision curve analysis (DCA) to determine the clinical practical value of the prediction model by quantifying the net benefit at different threshold probabilities [7]. It contains results that can theoretically tell us whether a model is worth using or which of several alternative models should be used. The closer the curve is to the upper right corner of the figure, the wider range of threshold probability that can generate net benefit is, and the higher the net benefit of the model is. All data analysis was performed by SPSS 26.0 (IBM, Armonk, NY, USA) statistical software or R language (R 4.0.4 version, http://www.r-project.org). The analysis results were obtained, and all statistical tests were statistically significant at *P* < 0.05.

## Results

### Demographic and clinicopathological characteristics

According to the inclusion and exclusion criteria, this study searched the SEER database for a total of 1079 patients from 2004 to 2015, and the clinicopathological characteristics mainly included age, sex, race, tumor site, tumor size, tumor depth, T stage, N stage, M stage, tumor grade, surgery, radiotherapy, and chemotherapy. The training cohort and validation cohort were randomized in a 7:3 ratio, and there was no significant difference in the demographic and clinicopathological characteristics between the two groups (*P* > 0.05). The baseline table is shown in Table 1.

**Table 1:** Demographic and clinicopathological characteristics of 1079 patients with UPS [n (%)]

Characteristic	Total (*n* = 1079)	Training (*n* = 755)	Validation (*n* = 324)	*P* value
Age/year				0.616
≤ 73	699 (64.8%)	485 (64.2%)	214 (66.0%)	
≥ 74	380 (35.2%)	270 (35.8%)	110 (34.0%)	
Sex				0.291
Female	465 (43.1%)	317 (42.0%)	148 (45.7%)	
Male	614 (56.9%)	438 (58.0%)	176 (54.3%)	
Race				0.316
White	897 (83.1%)	631 (83.6%)	266 (82.1%)	
Black	90 (8.4%)	57 (7.5%)	33 (10.2%)	
Other	92 (8.5%)	67 (8.9%)	25 (7.7%)	
Tumor site				0.431
Extremities	752 (69.7%)	524 (69.4%)	228 (70.4%)	
Trunk	258 (23.9%)	178 (23.6%)	80 (24.7%)	
Head/face/neck	69 (6.4%)	53 (7.0%)	16 (4.9%)	
Tumor size/cm				0.723
≤ 5.0	388 (36.0%)	274 (36.3%)	114 (35.2%)	
5.1–10.0	383 (35.5%)	269 (35.7%)	114 (35.2%)	
10.1–15.0	173 (16.0%)	115 (15.2%)	58 (17.9%)	
≥ 15.1	135 (12.5%)	97 (12.8%)	38 (11.7%)	
Extension				0.427
Superficial	269 (24.9%)	195 (25.8%)	74 (22.8%)	
Deep	620 (57.5%)	433 (57.4%)	187 (57.8%)	
NOS	190 (17.6%)	127 (16.8%)	63 (19.4%)	
T stage				0.781
T1	388 (36.0%)	274 (36.3%)	114 (35.2%)	
T2	691 (64.0%)	481 (63.7%)	210 (64.8%)	
N stage				0.966
N0	1047 (97.0%)	732 (97.0%)	315 (97.2%)	
N1	32 (3.0%)	23 (3.0%)	9 (2.8%)	
M stage				0.34
M0	1003 (93.0%)	706 (93.5%)	297 (91.7%)	
M1	76 (7.0%)	49 (6.5%)	27 (8.3%)	
Grade				0.726
I + II	141 (13.1%)	96 (12.7%)	45 (13.9%)	
III	350 (32.4%)	250 (33.1%)	100 (30.9%)	
IV	588 (54.5%)	409 (54.2%)	179 (55.2%)	
Surgery				0.194
No/unknown	63 (5.8%)	39 (5.2%)	24 (7.4%)	
Yes	1016 (94.2%)	716 (94.8%)	300 (92.6%)	
Radiation				0.231
No/unknown	380 (35.2%)	275 (36.4%)	105 (32.4%)	
Yes	699 (64.8%)	480 (63.6%)	219 (67.6%)	
Chemotherapy				0.709
No/unknown	865 (80.2%)	608 (80.5%)	257 (79.3%)	
Yes	214 (19.8%)	147 (19.5%)	67 (20.7%)	

### The selection of prognostic factor

#### Survival analysis

**Fig. 3:**
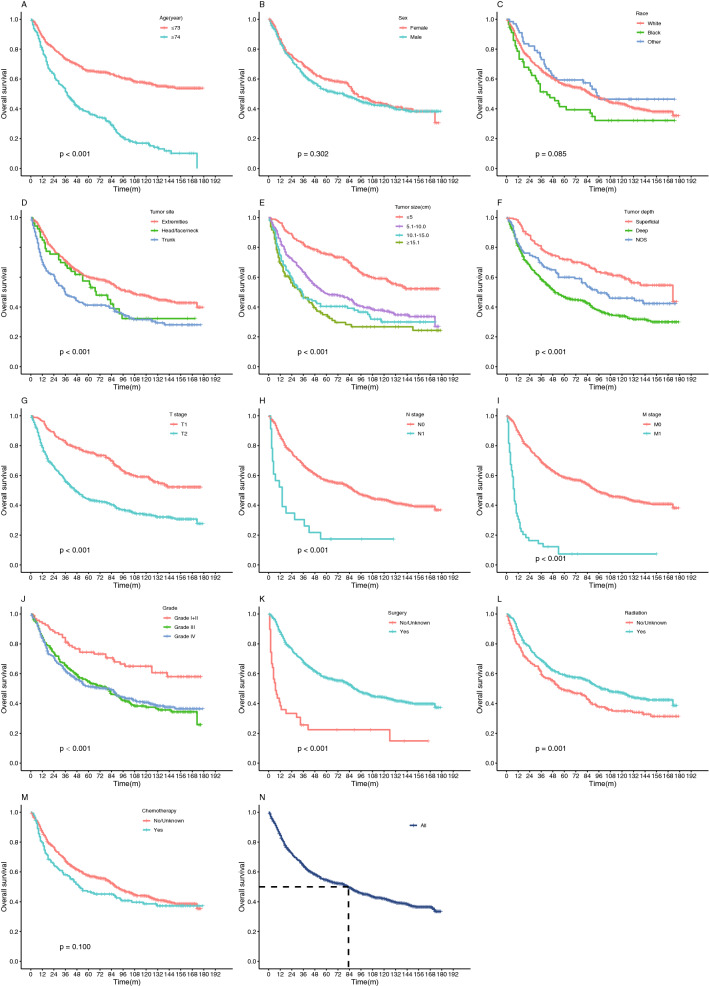
K–M survival curves of each variable on overall survival in patients with UPS. **A** Age, **B** sex, **C** race, **D** tumor site, **E** tumor size, **F** tumor depth, **G** T stage, **H** N stage, **I** M stage, **J** tumor grade, **K** surgery, **L** radiotherapy, **M** chemotherapy, **N** overall survival curves for 1079 UPS patients

In this study, the overall survival of patients with UPS was 83 months (Fig. 3), and the 3- and 5-year OS were 65.5% and 55.6%, respectively. The survival curves of each variable were made by the Kaplan–Meier method. The effects of each variable on the OS of UPS patients were compared and analyzed with the log-rank test. The results showed that the variables with statistically significant differences in the OS of patients were age, tumor site, tumor depth, tumor size, T stage, N stage, M stage, tumor grade, surgery, and radiotherapy. However, chemotherapy, sex and race had no statistically significant difference (*P* > 0.05). The K–M survival curves of each variable on overall survival in patients with UPS and overall survival K–M curves for 1079 UPS patients are shown in Fig. 3.

#### Univariate and multivariate Cox regression analysis

**Fig. 4:**
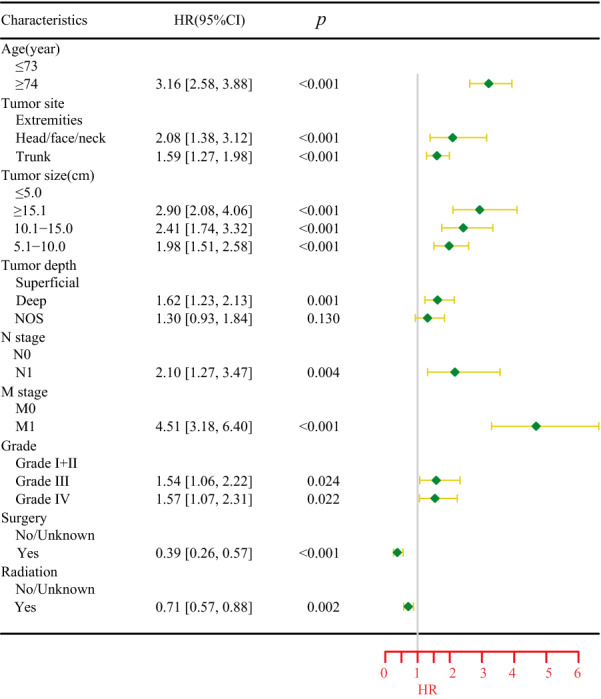
Cox regression analysis results of independent prognostic multivariate factors for OS in patients

T stage is mainly determined by tumor size and tumor depth. In order to compare the effect of different sizes of tumors on the prognosis of patients in more detail, tumor size and tumor depth were used instead of T stage in Cox regression analysis. The results were consistent with the K–M survival curve results by univariate Cox regression analysis of the training cohort. The prognostic factors with statistical differences in univariate Cox regression analysis were further analyzed by multivariate Cox regression analysis, and the results determined the independent influencing factors of age, tumor site, tumor size, tumor depth, N stage, M stage, tumor grade, surgery and radiotherapy. Univariate and multivariate Cox regression analysis are shown in Table 2 and Fig. 4.

**Table 2:** Cox regression analysis results of independent prognostic univariate factors for OS in patients with UPS

Characteristic	Univariate		
	*Wald χ*^*2*^	*P*	HR[95%CI]
Age/year			
≤ 73	Ref.		
≥ 74	104.87	< 0.001	2.80[2.30–3.40]
Sex			
Female	Ref.		
Male	1.06	0.303	1.11[0.91–1.35]
Race			
White	Ref.		
Black	3.44	0.064	1.38[0.98–1.95]
Other	1.01	0.313	0.83[0.58–1.19]
Tumor site			
Extremities	Ref.		
Trunk	24.50	< 0.001	1.73[1.39–2.15]
Head/face/neck	2.37	0.124	1.34[0.92–1.95]
Tumor size/cm			
≤ 5.0	Ref.		
5.1–10.0	29.54	< 0.001	1.98[1.55–2.53]
10.1–15.0	37.04	< 0.001	2.53[1.88–3.41]
≥ 15.1	48.78	< 0.001	2.98[2.20–4.06]
Extension			
Superficial	Ref.		
Deep	34.31	< 0.001	2.16[1.67–2.80]
NOS	6.25	0.012	1.53[1.10–2.13]
N stage			
N0	Ref.		
N1	25.32	< 0.001	3.28[2.06–5.20]
M stage			
M0	Ref.		
M1	127.51	< 0.001	6.25[4.54–8.58]
Grade			
I + II	Ref.		
III	14.26	< 0.001	2.05[1.41–2.98]
IV	14.98	< 0.001	2.04[1.42–2.92]
Surgery			
No/unknown	Ref.		
Yes	43.82	< 0.001	0.29[0.20–0.42]
Radiation			
No/unknown	Ref.		
Yes	10.84	0.001	0.72[0.59–0.87]
Chemotherapy			
No/unknown	Ref.		
Yes	2.66	0.103	1.22[0.96–1.54]

#### Construction and validation of nomogram

**Fig. 5:**
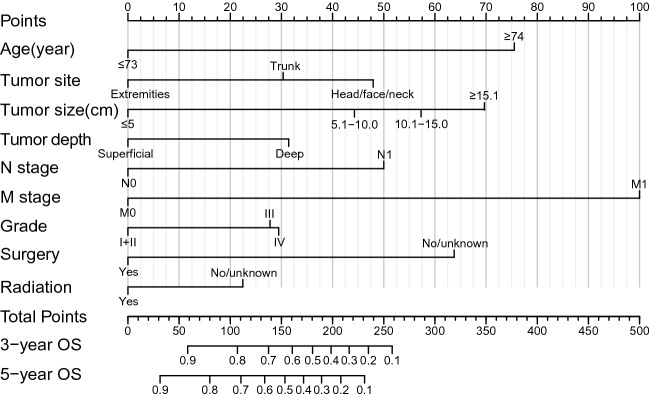
Predicting 3-year and 5-year overall survival nomogram prediction models for patients with UPS

A nomogram was developed based on nine variables: age, tumor site, tumor size, tumor depth, N stage, M stage, tumor grade, surgery, and radiotherapy through the "rms" package of R language. Nomogram is shown in Fig. 5. The scores for each factor are shown in Table 3.

**Table 3:** Corresponding score of each factor in nomogram

Characteristics	Points OS
Age (year)	
≤ 73	0
≥ 74	76
Tumor site	
Extremities	0
Trunk	30
Head/face/neck	48
Tumor size (cm)	
≤ 5.0	0
5.1–10.0	44
10.1–15.0	57
≥ 15.1	70
Tumor depth	
Superficial	0
NOS	17
Deep	31
N stage	
N0	0
N1	50
M stage	
M0	0
M1	100
Grade	
I + II	0
III	28
IV	29
Surgery	
No	64
Yes	0
Radiation	
No	22
Yes	0

The nomogram were validated internally and externally by the training cohort and validation cohort, respectively. The C-index of training cohort was 0.756 (95%CI: 0.731–0.780), and the C-index of validation cohort was 0.757 (95%CI: 0.722–0.791), which were both higher than the C-index of the TNM staging system, respectively, which was 0.673 (95%CI: 0.647–0.698).

**Fig. 6:**
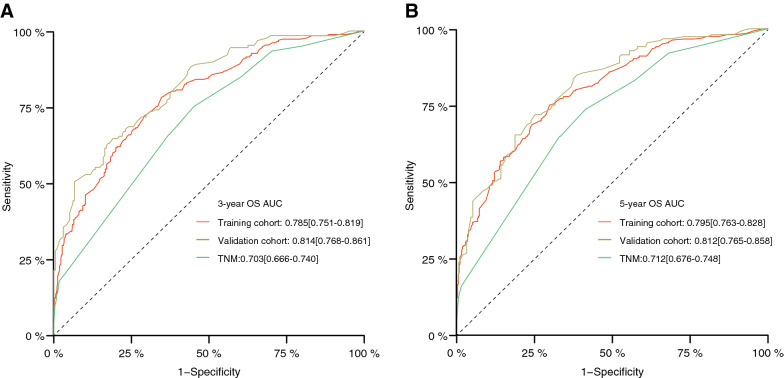
ROC curves and AUC values for training cohort and validation TNM staging system. Sensitivity: true positive rate; 1-specificity: false positive rate

The AUC of nomogram of 3-year and 5-year OS in the training cohort were 0.785 and 0.795, respectively; the AUC of nomogram of 3-year and 5-year OS in the validation cohort were 0.814 and 0.812, respectively; and the AUC of TNM staging system of 3-year and 5-year OS were 0.703 and 0.712, respectively, which were lower than those of the nomogram. The ROC curves and AUC values for each group are shown in Fig. 6.

**Fig. 7:**
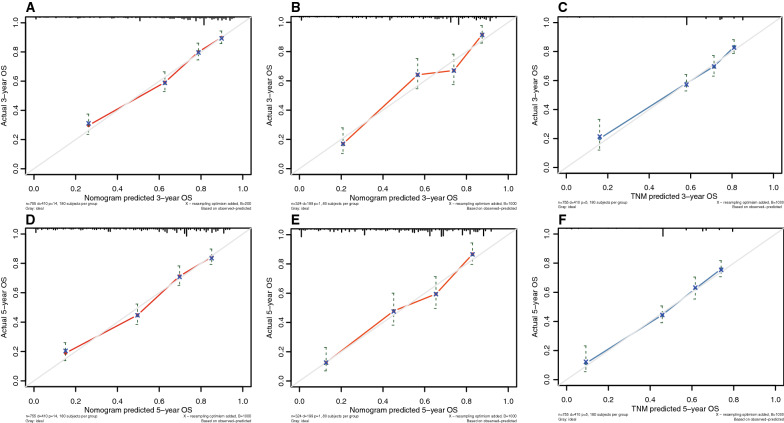
Nomogram and calibration curve of TNM staging system. **A** and **D**: training cohort; **B** and **E**: validation cohort; **C** and **F**: TNM staging system

The calibration curves of nomogram and TNM staging system for predicting 3-year and 5-year overall survival rate in patients with UPS are shown in Fig. 7. According to the calibration curve, it can be seen that the calibration curve of the nomogram and TNM staging system and 45° line had a good degree of fit.

**Fig. 8:**
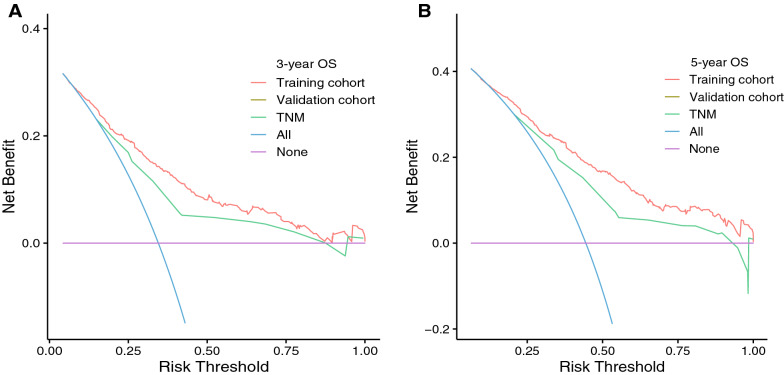
Decision curve analysis of the nomogram and the TNM staging system. **A** and **B**: the training cohort; **C** and **D**: validation cohort

Decision curve analysis (DCA) determines the clinical value of a predictive model by quantifying the net benefit at different threshold probabilities [8]. The abscissa represents the threshold probability and the ordinate represents the net gain. The x-axis (purple line) indicates that no patient experienced an outcome event and the net benefit is zero. The diagonal line (blue line) indicates that all patients have outcome events. The red line and green line in Fig. 8 are the decision analysis curves of nomogram and TNM staging system, respectively. The closer the curve is to the upper right corner of the figure, the wider the range of threshold probability that can produce net benefit, and the higher net benefit of the model is. The results of decision curve analysis showed that the threshold probability range of net benefit generated by nomogram was wider than that of TNM staging system, and the nomogram had higher net benefit than TNM staging system as a whole.

## Discussion

UPS is a more common and aggressive type of tumor in STS and tends to occur in aged people, mostly males. It typically presents as asymptomatic, insignificant, and rapidly growing skin or subcutaneous nodules without superficial skin abnormalities [9]. UPS forms mainly in the extremities and trunk, secondly in the head and neck region, retroperitoneum, bones, and other organs [9, 10]. In almost half of the cases, the sites of tumors are located in the skin and subcutaneous soft tissues [11, 12], with deep soft tissue being more common. Therefore, UPS is usually diagnosed late, and prone to local recurrence and metastasis after surgery. However, the results vary greatly between different studies, with local recurrence rate (LRR) ranging from 14 to 75%, distant metastasis rate ranging from 7 to 38%, and 5-year overall survival rate ranging from 53 to 68%[9, 13–18], manifesting a relatively poor overall prognosis [18]. In this study, the 3-year and 5-year CSS of patients with UPS are 71.4% and 64%, respectively, and the 3-year and 5-year OS of patients with UPS are 65.5% and 55.6%, showing a similar results to the previous studies [9, 13–18].

Patients with UPS are predominantly the elderly [11]). In this study, age was an independent prognostic factor that affected the patients with UPS. The prognosis of aged patients was generally poor, and the risk of death increased significantly as age grew. This may be related to the fact that elder patients often have comorbidities and respond inactively to the treatment. In addition, gender and race factors had no significant effect on the prognosis of patients with UPS, which is consistent with the previous findings [19, 20].

The primary site of UPS was mainly the extremities, followed by the trunk position, and occurred less frequently in the head and neck region [21, 22] and head and neck UPS were more aggressive and had a poorer prognosis [10, 23], which was consistent with previous research. This may be due to the fact that there were many important structures in the head and neck region. The local vascular supply and lymphatic network in the face and neck were rich. Surgical resection to preserve vital organs and functions may lead to incomplete tumor resection and increase the postoperative recurrence rate [24–26]. For patients with UPS, tumor size was also an important factor affecting the prognosis. The larger tumor size was, the worse prognosis of patients with tumors. This may be due to the fact that larger tumors undergo more cell division cycles than smaller tumors, allowing variant tumors with metastatic capacity to grow adequately. While tumor growth is associated with cell cycle dysregulation or tumor angiogenesis, tumors are aggressive and more likely to develop distant metastasis, so patients have a poor prognosis [14, 16, 27]. At the same time, when the UPS originates or extends below the subcutaneous fat, it has aggressive behavior and an increased risk of metastasis and death [10, 16, 21].

Tumor grade reflects the morphological differences between tumor tissues and normal tissue cells in terms of tissue structure and cell morphology, and can well reflect the biological characteristics and metastatic potential of tumors. High-grade tumors have a poor prognosis, which was consistent with the results of this study. Lymph node metastasis and distant metastasis are important characteristics of advanced tumor stage and tumor progression, and also important factors that affected the prognosis of patients [9, 19]. In this study, the overall mortality risk of UPS patients with lymph node metastasis was 2.10 times higher than the patients without lymph node metastasis, respectively. The corresponding risks of death in the patients with distant metastasis were 4.79 and 4.51 times higher than that in the patients without distant metastasis. The above observation indicates that the patients with tumor metastasis had a worse prognosis, and the survival rate of the patients with distant metastasis had a significant decrease.

Currently, surgery is still the basic treatment for UPS and a key measure to prevent tumor recurrence and metastasis. Besides, radiotherapy also plays an essential role in the treatment of local and metastatic STS [28]. The results of this study show that for patients with UPS, surgery reduced the risk of cancer-specific death by 59% and the overall risk of death by 61%, and radiotherapy reduced the risk of cancer-specific death by 24% and the overall risk of death by 29%, demonstrating that surgery and radiotherapy can improve the prognosis of UPS patients to some extent. However, chemotherapy had no significant effect on OS in patients with UPS, which is consistent with previous findings [3, 9, 19, 29]. Therefore, chemotherapy is not used as the first choice of treatment for patients with UPS much, but as an alternative when the patient is inoperable or the tumor spreads and metastasizes.

By applying the K–M method and Cox regression analysis, this study determined nine independent prognostic factors: age, tumor site, tumor size, tumor depth, N stage, M stage, tumor grade, surgery, and radiotherapy. Based on the selected prognostic factors, the study constructed nomograms for predicting the 3-year and 5-year OS of UPS patients, respectively. In order to avoid the over-interpretation of the model data and confirm its clinical usefulness, the validation of the nomograms is crucial [4]. In the predictive model of this study, both the modeling cohort and the validation cohort had a C-index > 0.75 and an AUC > 0.78, while were higher than that of the TNM staging system, indicating that the nomogram had better discrimination in predicting prognosis. The calibration curves of the training cohort, validation cohort, and the TNM staging system were well fitted with the 45° line, which supported that the values of the predicted survival rate in the nomograms and the TNM staging system were close to patients' actual survival rate. Given that there existed a good consistency between the nomograms and the TNM staging system, both of which had an accurate predictive ability. The decision curve analysis showed that compared with the TNM staging system, the nomograms could generate clinical net benefit with a wider threshold range, and its overall net benefit was also greater than that of the TNM staging system, indicating that the nomogram had better clinical usefulness.

The SEER database is an authoritative source of information on cancer incidence and survival that collects data from multiple cancer registries on cancer diagnosis, treatment, and survival [30]. Its high quality, multi-center and large-sample data make the clinical study based on SEER database have high clinical reference value. This study also has some limitations that should be noted. Firstly, our study is a retrospective study. Due to the lack of information, many cases were excluded, causing a selection bias. Secondly, because of the limitations of the SEER database, some prognostic factors that were not included in our study such as family history, receipt of targeted therapy or immunotherapy, and recurrence of the tumor, may have affected our results [31]. Finally, the patient information in the SEER database comes from the United States, which may lack representativeness on a global scale [32].Therefore, the study on the treatment and prognosis of patients with UPS still need to be supported by the results of further large randomized controlled trials.

In summary, the study included age, tumor site, tumor size, tumor depth, N stage, M stage, tumor grade, surgery, and radiotherapy in the nomogram. And through discrimination, calibration, and decision curve analysis, it demonstrated the nomogram's strong discriminatory ability, accuracy, and clinical practicability to the survival prediction, indicating that the nomogram had a good predictive ability for 3-year and 5-year OS of UPS patients. This has important clinical significance for clinicians to reduce or enhance the intensity of follow-up monitoring, better communicate with patients, and provide personalized clinical information for patients.

## Conclusion

Age, tumor site, tumor size, tumor depth, N stage, M stage, Grade, surgery and radiotherapy are independent prognostic factors for patients with undifferentiated pleomorphic sarcoma. Among them, surgery and radiotherapy can improve the prognosis of patients to a certain extent. No effect of sex, race and chemotherapy on prognosis was found. The nomogram can individually predict 3-year and 5-year OS of UPS patients, with good discrimination, accuracy and clinical utility. It can provide a reference for clinicians and patients to make better clinical decisions.

## Data Availability

The datasets generated and/or analyzed during the current study are available in the Surveillance, Epidemiology, and End Results (SEER) database repository, https://seer.cancer.gov.

## Abbreviations

UPS: Undifferentiated pleomorphic sarcoma
STS: Soft tissue sarcoma
OS: Overall survival
CSS: Cancer-specific survival
SEER: Surveillance, Epidemiology, and End Results
AJCC: American Joint Committee on Cancer
C-index: Concordance index
ROC: Receiver operating characteristic
AUC: Area under curve
DCA: Decision curve analysis
HR: Hazard ratio
CI: Confidence interval
